# Marked Antigenic Divergence and Evolutionary Analysis of H5 AIVs from Wild Birds in East China, 2013–2022

**DOI:** 10.3390/ani16132109

**Published:** 2026-07-07

**Authors:** Xiang Su, Keyu Cai, Yuhan Zong, Yunfei Guo, Yuncong Yin, Xian Zheng, Xinyu Miao, Hui Yang, Tao Qin, Daxin Peng, Sujuan Chen

**Affiliations:** 1 College of Veterinary Medicine, Yangzhou University, Yangzhou 225000, China; dx120210177@stu.yzu.edu.cn (X.S.); 242001102@stu.yzu.edu.cn (K.C.); 242001179@stu.yzu.edu.cn (Y.Z.); mz120231630@stu.yzu.edu.cn (Y.G.); yzyuncong@hotmail.com (Y.Y.); mx120230981@stu.yzu.edu.cn (X.Z.); miaoxy@yzu.edu.cn (X.M.); 008689@yzu.edu.cn (H.Y.); qintao@yzu.edu.cn (T.Q.); 2 Jiangsu Co-Innovation Center for the Prevention and Control of Important Animal Infectious Disease and Zoonoses, Yangzhou 225009, China; 3 Jiangsu Research Centre of Engineering and Technology for Prevention and Control of Poultry Disease, Yangzhou 225009, China

**Keywords:** avian influenza virus, H5 subtype, wild birds, genetic evolution, antigenic difference

## Abstract

The highly pathogenic H5 subtype avian influenza viruses (AIVs) pose persistent threats to the poultry industry and public health owing to their high lethality and pandemic potential. Migratory wild birds play a pivotal role in the global dissemination and genetic reassortment of the virus, serving as both natural reservoirs and long-distance vectors that drive its spatiotemporal spread. However, the extent and evolutionary drivers of antigenic divergence among H5 AIVs circulating in wild birds in East China remain poorly understood. Here, we aim to characterize the evolutionary dynamics and antigenic divergence of H5 AIVs isolated from wild birds in East China between 2013 and 2022. Whole-genome sequencing and phylogenetic analysis revealed that the isolates belonged to multiple clades, including 2.3.2.1 and 2.3.4.4, and encompassed the H5N1, H5N6, and H5N8 subtypes. Key amino acid site analysis showed that the glycosylation site patterns in the HA and NA proteins varied among clades, with some strains exhibiting gains or losses of glycosylation sites, while certain strains had acquired mutations associated with mammalian adaptation. Cross-hemagglutination inhibition (HI) assays combined with antigenic cartography demonstrated that the majority of the isolates were antigenically well-matched with the contemporaneous vaccine strains used in China, indicating that these vaccines effectively covered the predominant circulating antigenic variants at the time. Nevertheless, potential antigenic mismatches were still observed between some circulating strains and these vaccine strains. These findings suggest that wild birds in East China may contribute to the regional movement and diversification of H5 AIVs, highlighting the value of sustained surveillance for early warning and vaccine strain evaluation.

## Simple Summary

Wild birds serve as reservoirs and vectors, playing a critical role in the evolution and spread of H5 avian influenza viruses. Eastern China lies along the East Asian–Australasian Flyway, providing important stopover and wintering sites, while intensive poultry farming and live poultry markets increase contact between wild and domestic poultry. From 2013 to 2022, 16 H5 viruses isolated from wild birds in eastern China were purified, sequenced, and analyzed. These viruses exhibited considerable genetic diversity and carried mammalian adaptation mutations. Most isolates matched the vaccine strains used in China at the time, but potential antigenic mismatches remained. Continuous surveillance in wild birds can therefore provide early warning for domestic poultry, enabling timely detection of variants and vaccine updates.

## 1. Introduction

Avian influenza virus (AIV), a member of the family Orthomyxoviridae, is a single-stranded, negative-sense RNA virus with a genome composed of eight segments. Based on antigenic differences in the surface glycoproteins hemagglutinin (HA) and neuraminidase (NA), AIVs are currently classified into 18 HA subtypes (H1-H18) and 11 NA subtypes (N1-N11) [1]. Among these, H5 subtype avian influenza viruses, particularly highly pathogenic avian influenza viruses (HPAIVs), pose a serious threat to the poultry industry and can cross the species barrier to infect mammals, including humans [2], thereby presenting a persistent challenge to public health security [3]. Since the first isolation of the A/goose/Guangdong/1/96 (Gs/GD/96) H5N1 virus from geese in Guangdong, China, in 1996, viruses of this lineage have continuously evolved into multiple clades and, through reassortment, have generated various subtypes such as H5Nx, which have gradually spread to many regions worldwide [4,5].

Wild birds are recognized as the natural reservoir of avian influenza viruses. In particular, species belonging to the orders Anseriformes and Charadriiformes play critical roles in the long-term maintenance, transmission, and evolution of these viruses [6]. The migratory behavior of wild birds facilitates the long-distance dissemination of avian influenza viruses across geographical regions, thereby promoting transboundary transmission and genetic reassortment [7]. East China, located at a key node of the East Asian–Australasian Flyway, contains abundant wetland resources and diverse wild bird habitats, making it a crucial region where wild bird migration intersects with poultry production. These ecological conditions provide an ideal setting for the spread, reassortment, and mutation of H5 subtype avian influenza viruses [8]. In recent years, frequent outbreaks of H5 subtype avian influenza viruses in wild birds have suggested that these viruses have established sustained transmission cycles within wild bird populations [9].

Although H5 subtype avian influenza viruses of poultry origin have been extensively studied, long-term systematic surveillance data on the genetic evolution and antigenic variation of H5 viruses in wild birds in East China remain limited. In this study, H5 AIVs isolated from wild birds in East China during 2013–2022 were characterized through whole-genome sequencing, phylogenetic analysis, and antigenic profiling. We hypothesized that these viruses would exhibit genetic heterogeneity, evidence of reassortment, and variable degrees of antigenic relatedness to vaccine strains deployed in China during different periods. This study aimed to elucidate the transmission dynamics and molecular evolutionary trends of H5 AIVs in wild bird populations, thereby providing a scientific basis for early warning of avian influenza outbreaks, vaccine strain selection, and the formulation of region-specific prevention and control strategies.

## 2. Materials and Methods

### 2.1. Sample Collection and Virus Isolation

From 2013 to 2022, a total of 27,000 samples were collected from key freshwater lakes and natural wetlands along the East Asian–Australasian Flyway in East China (a detailed breakdown by sampling location and year is provided in Supplementary Table S1). The sampled species spanned multiple orders, predominantly Anseriformes (*Tadorna ferruginea*, *Anas platyrhynchos*, *Mergus merganser*), Charadriiformes (*Larus ridibundus*, *Larus argentatus*, *Himantopus himantopus*), and other waterbirds (*Egretta garzetta*, *Ardea cinerea*, *Grus grus*, *Grus japonensis*, etc.). After collection, swab samples were placed in viral transport medium, stored in a portable cooler at 4 °C, transported to the laboratory within 24 h, and immediately stored at −80 °C until use. Before virus isolation, the swab samples in viral transport medium were thoroughly mixed, and solid debris was removed by centrifugation at 10,000× *g* for 10 min. The resulting supernatant was inoculated into 10-day-old specific-pathogen-free (SPF) embryonated chicken eggs at a dose of 0.2 mL per egg, with three eggs used per strain. The inoculated eggs were then incubated at 37 °C for 96 h.

### 2.2. Plaque Purification of Virus

To ensure genetic homogeneity, three rounds of plaque purification were performed for all 16 isolates in MDCK cells. MDCK cells (ATCC, Manassas, VA, USA; CCL-34) were cultured in Dulbecco’s modified Eagle’s medium (DMEM; Procell, Wuhan, China) supplemented with 10% fetal bovine serum (Gibco, Waltham, MA, USA) at 37 °C under 5% CO_2_ and seeded into 6-well plates (Corning Incorporated, Corning, NY, USA). Once the cells had formed confluent monolayers, the virus was serially diluted 10-fold in serum-free DMEM and inoculated onto the monolayers. After 1 h of adsorption, the cells were overlaid with a 1:1 mixture of 1.5% agar and 2× high-glucose DMEM (Genom, Hangzhou, China) containing TPCK-treated trypsin (Sigma, St. Louis, MO, USA) at a final concentration of 1 μg/mL. The plates were inverted and incubated at 37 °C with 5% CO_2_ for 72 h, and plaque formation was monitored daily. Subsequently, 0.33% neutral red staining solution (Sigma, St. Louis, MO, USA) was diluted 1:20 in PBS and applied to the agar overlay. After an additional 12 h of inverted incubation, the plaques were picked from each purification round. The picked plaques were subjected to three freeze–thaw cycles, propagated in 10-day-old specific-pathogen-free (SPF) embryonated chicken eggs, and the resulting viral stocks were stored at −80 °C.

### 2.3. Whole-Genome Sequencing

Viral RNA was extracted from allantoic fluid using TRIzol reagent (Invitrogen, Carlsbad, CA, USA) according to the manufacturer’s instructions and reverse-transcribed into cDNA, which was subsequently used as the template for PCR amplification. All gene segments were amplified using Hoffman primers [10] and sequenced by GenScript Biotech Corporation. The complete genome sequences of all isolates generated in this study were deposited in GenBank.

### 2.4. Phylogenetic Analysis

To accurately genotype the viruses and clarify evolutionary relationships, reference sequences were objectively selected based on WHO/WOAH-recommended representative strains and NCBI BLASTN 2.17.0+ homology searches, in addition to the 16 isolates from this study. Sequences were assembled using SnapGene 6.0.2, and phylogenetic analysis was performed with PhyloSuite v1.2.2. The best-fit substitution model was selected using ModelFinder. Maximum-likelihood (ML) phylogenetic trees were then constructed using IQ-TREE, employing the ultrafast bootstrap approximation with 10,000 replicates, a maximum of 1000 iterations, and a minimum correlation coefficient of 0.90. The resulting trees were visualized using the online tool iTOL (https://itol.embl.de/, accessed on 11 April 2026).

### 2.5. Molecular Characterization

Molecular characterization was performed using MegAlign pro 17.1 and MEGA X to analyze key protein features and mutation sites. Potential glycosylation sites in the HA and NA proteins were predicted using the online bioinformatics tool NetNGlyc 1.0 Server (https://services.healthtech.dtu.dk/services/NetNGlyc-1.0/, accessed on 7 April 2026).

### 2.6. Selection Pressure Analysis

To evaluate selection pressure on each gene segment, sequences were first aligned using MAFFT, after which stop codons were removed and the aligned sequences were exported in FASTA format. The best-fit substitution model was selected using the Datamonkey web server (http://www.datamonkey.org/, accessed on 3 May 2026). Positive selection sites in the eight gene segments of the 16 isolates were analyzed using four algorithms: fixed effects likelihood (FEL), mixed effects model of evolution (MEME), fast unconstrained Bayesian approximation (FUBAR), and single-likelihood ancestor counting (SLAC). The selection coefficient ω represents the ratio of nonsynonymous to synonymous substitution rates (dN/dS), where ω > 1 indicates positive selection, ω = 1 indicates neutral evolution, and ω < 1 indicates negative (purifying) selection.

### 2.7. Antigenic Analysis

Antigenic differences among the isolates were analyzed using cross-hemagglutination inhibition (HI) assays in accordance with World Health Organization standard operating procedures. Phylogenetic analysis of the HA gene grouped the 16 isolates into six well-supported clades. To represent the full genetic diversity of the collection without testing highly similar strains redundantly, genetically distant strains were selected from each clade (JYWB4, QP10, GY183, GY999, SSW7, CM120, SH17, DT10, and CIXI20). The nine strains were subsequently used to prepare inactivated oil-emulsion vaccines. For each strain, a group of five 6-week-old SPF chickens (*n* = 5) was immunized with 0.4 mL of the corresponding vaccine per bird. The vaccines were prepared by emulsifying inactivated whole virus with white oil and Tween-80 adjuvant using a homogenizer. Once satisfactory serum antibody titers had been achieved, serum samples from each group were collected, pooled, and subjected to cross-HI assays. HI assays were performed according to the standard protocol [11]. Viruses were diluted to 4 hemagglutination units (HAU) per 25 μL. In a 96-well V-bottom microtiter plate, 25 μL PBS was added to each well, followed by 25 μL of test serum in the first well. Serial twofold dilutions of serum were made, and then 25 μL of 4-HAU antigen was added to all wells. After incubation at 37 °C for 15 min, 25 μL of 1% chicken red blood cell suspension was added, mixed, and the plate incubated for an additional 15 min before recording the hemagglutination inhibition endpoints. The highest serum dilution that yielded a button-like pellet of erythrocytes was defined as the HI titer. All HI assays were performed in three independent experiments. The resulting HI titers were further analyzed via antigenic cartography (https://www.antigenic-cartography.org/, accessed on 8 April 2026).

## 3. Results

### 3.1. Prevalence and Sequencing of H5 Subtype AIV

**Figure 1:**
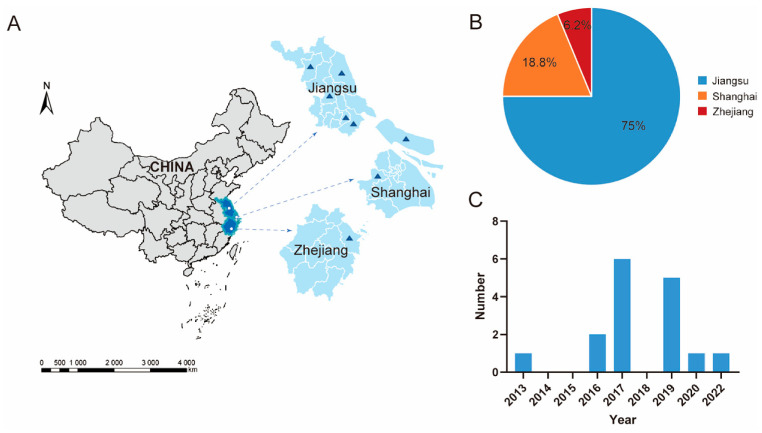
Surveillance profile of H5 AIV isolates from Wild birds in East China during 2013–2022. (**A**) Distribution of sampling sites. Blue: the sampled provinces; triangle: the specific sampling sites; (**B**) Virus isolation rates in different sampling regions; (**C**) Temporal distribution and number of isolated strains.

From 2013 to 2022, major freshwater lakes and natural wetlands in East China were selected as sampling sites (Figure 1A), including Lake Taihu, Lake Gaoyou, and Lake Hongze in Jiangsu Province, Hangzhou Bay in Zhejiang Province, and Chongming Island in Shanghai. A total of 27,000 fecal and environmental samples were collected from wild bird habitats. Virus isolation and identification yielded 16 H5 subtype AIVs in 2013, 2016, 2017, 2019, 2020, and 2022 (Table 1; Supplementary Table S2), with an overall isolation rate of 0.6‰. Among these isolates, 12 were obtained from Jiangsu Province, accounting for 75.0% of all positive isolates, whereas three were from Shanghai (18.8%) and one was from Zhejiang Province (6.2%) (Figure 1B,C). After three rounds of plaque purification, the complete genome sequences of all isolates were obtained.

### 3.2. Phylogenetic Relationships

#### 3.2.1. HA Gene

**Figure 2:**
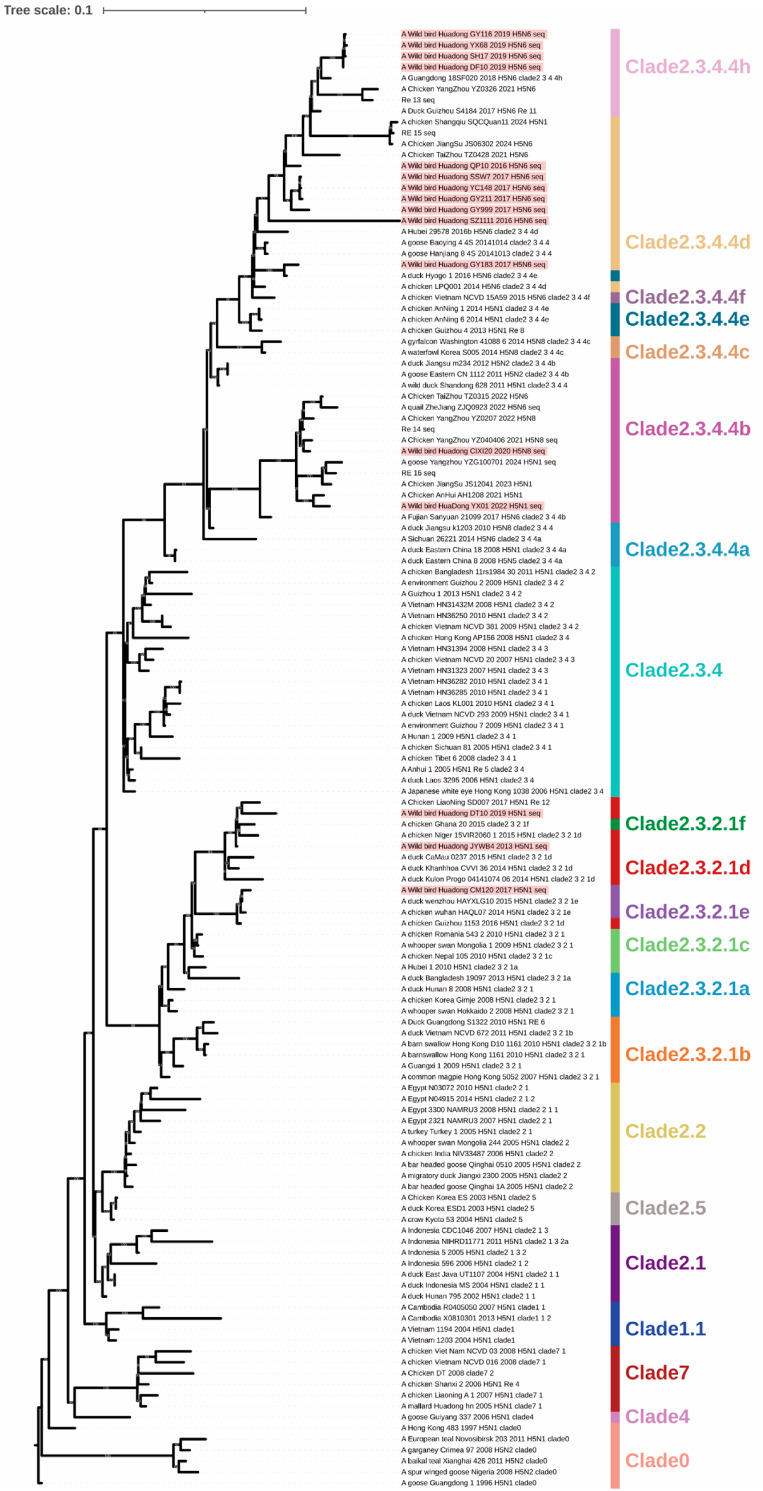
Phylogenetic tree based on the HA gene sequences of 16 H5 AIV isolates from wild birds in East China, 2013–2022. The isolates are highlighted with a pink background.

Phylogenetic analysis of the HA gene showed that, among the H5N1 subtype AIVs, CM120 belonged to clade 2.3.2.1e, whereas JYWB4 and DT10 belonged to clade 2.3.2.1d; notably, DT10 was genetically closely related to the Re-12 vaccine strain. YX01 belonged to clade 2.3.4.4b and was genetically closely related to the Re-16 vaccine strain. Among the H5N6 subtype AIVs, GY183 belonged to clade 2.3.4.4e; GY999, GY211, SSW7, YC148, QP10, and SZ1111 belonged to clade 2.3.4.4d; and DF10, GY116, SH17, and YX68 belonged to clade 2.3.4.4h and were genetically closely related to the Re-13 vaccine strain. The H5N8 isolate CIXI20 belonged to clade 2.3.4.4b and was genetically closely related to the Re-14 vaccine strain (Figure 2).

#### 3.2.2. NA Gene

**Figure 3:**
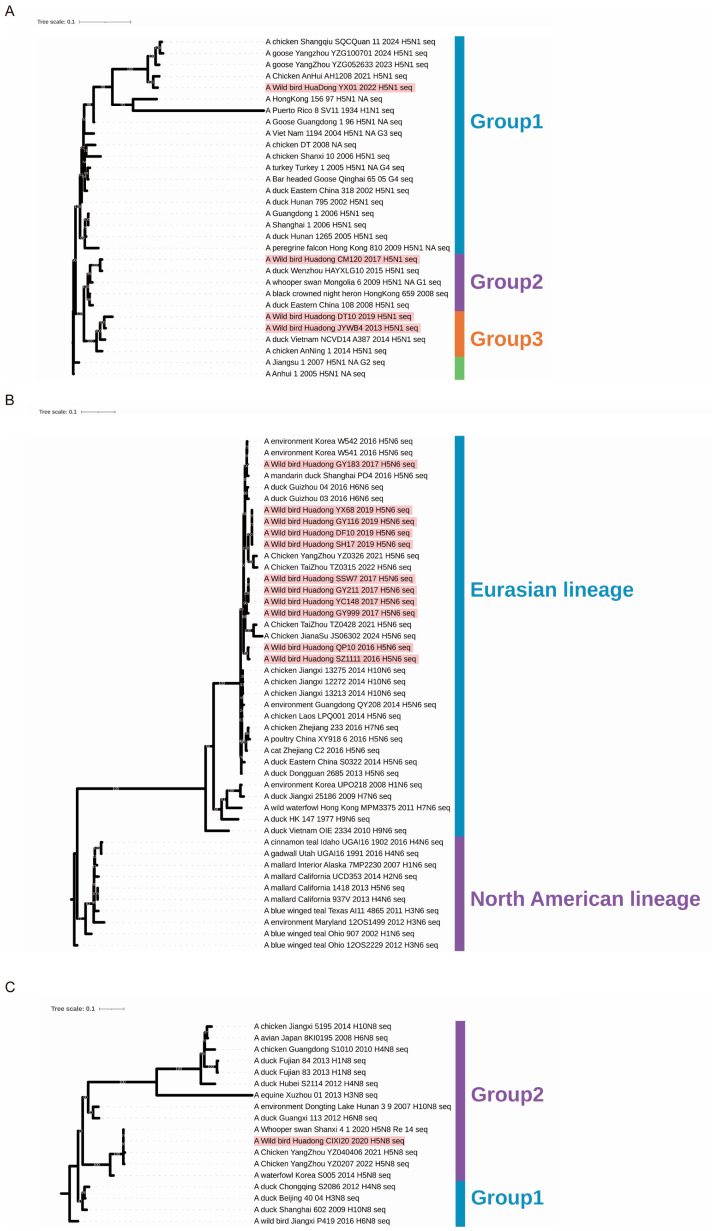
Phylogenetic tree based on the NA gene sequences of 16 H5 AIV isolates from wild birds in East China, 2013–2022. (**A**) Phylogenetic tree of the NA gene of H5N1 subtype AIVs; (**B**) Phylogenetic tree of the NA gene of H5N6 subtype AIVs; (**C**) Phylogenetic tree of the NA gene of H5N8 subtype AIVs. The isolates are highlighted with a pink background.

Phylogenetic analysis of the NA gene showed that the isolates comprised four H5N1 strains, eleven H5N6 strains, and one H5N8 strain. The NA genes of the four H5N1 viruses were distributed among groups 1–3, with YX01 being genetically distinct from the other strains. Group 1 evolved from the Gs/GD/96 lineage, whereas groups 2 and 3 represented more recently circulating N1 clades (Figure 3A). Phylogenetic analysis of the N6 gene indicated that all eleven isolates belonged to the Eurasian lineage and were genetically distant from the North American lineage (Figure 3B). Analysis of the N8 gene showed that the NA gene of CIXI20 was genetically closely related to that of the Re-14 vaccine strain (Figure 3C).

#### 3.2.3. PB2 Gene

**Figure 4:**
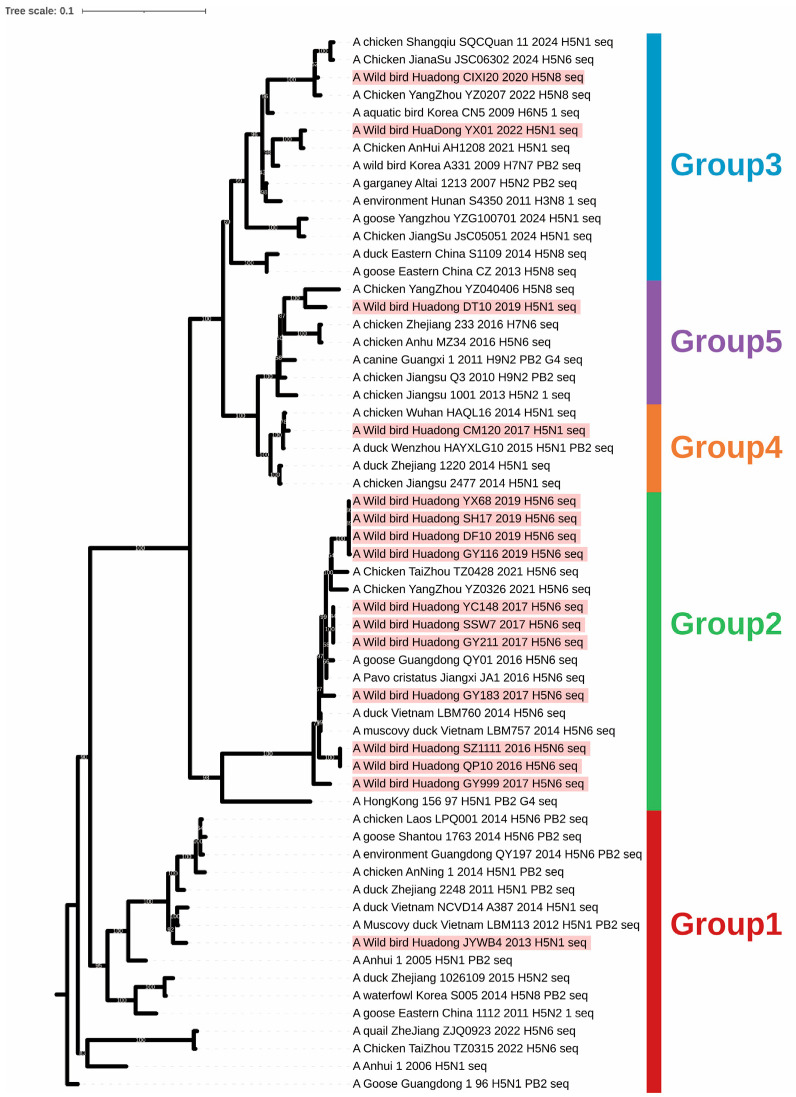
Phylogenetic tree based on the PB2 gene sequences of 16 H5 AIV isolates from wild birds in East China, 2013–2022. The isolates are highlighted with a pink background.

The PB2 genes of the H5 subtype AIVs were mainly distributed among five phylogenetic clades (Figure 4). The PB2 gene of JYWB4 was located in Group 1 and originated from an H5N1 subtype strain (A/goose/GuangDong/1/96); the PB2 genes of the eleven H5N6 subtype AIVs were all located in Group 2 and originated from A/HongKong/156/97; the PB2 gene of CM120 was located in Group 4 (which mainly originated from an H5N1 subtype strain, A/duck/WenZhou/HAYXLG10/15) and was closely related to the current Re-10 epidemic strain in China.

#### 3.2.4. PB1 Gene

**Figure 5:**
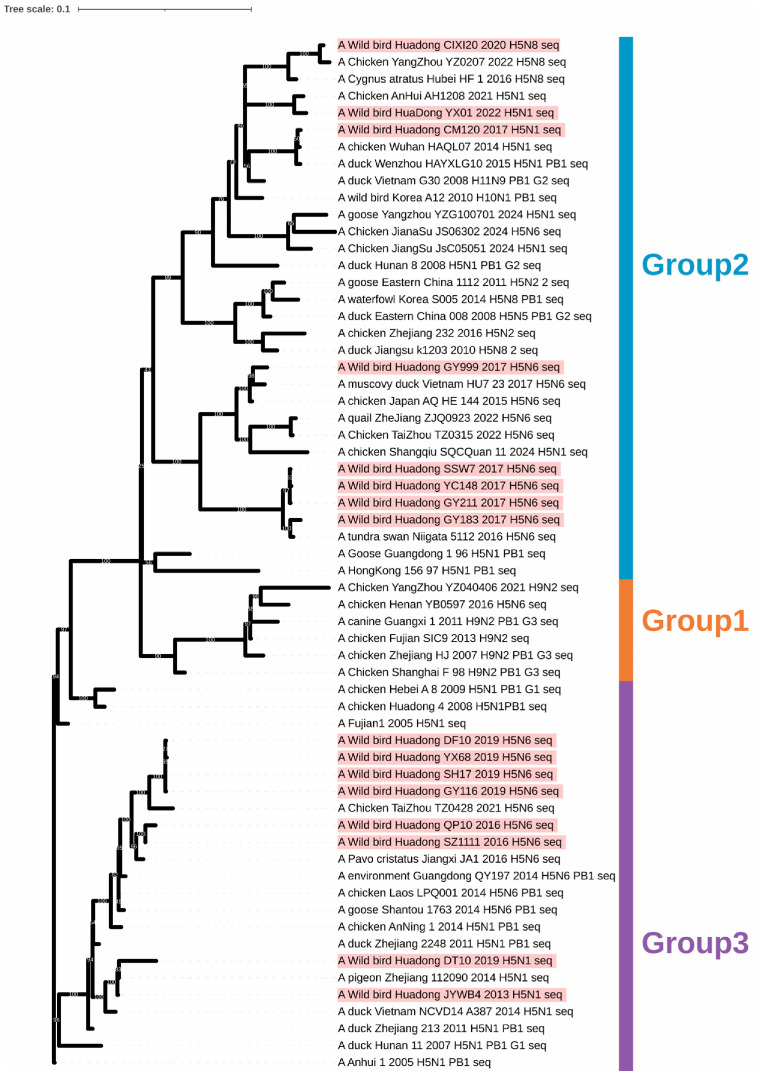
Phylogenetic tree based on the PB1 gene sequences of 16 H5 AIV isolates from wild birds in East China, 2013–2022. The isolates are highlighted with a pink background.

The phylogenetic tree of the PB1 genes (Figure 5) showed that the isolates fell into Groups 2 and 3. Group 2 originated from A/Goose/Guangdong/1/96 and contained most of the clade 2.3.4.4b and 2.3.4.4d isolates. Group 3 contained the Re-5 vaccine strain A/Anhui/1/2005 and the prevalent clade 2.3.4.4d strain A/chicken/LPQ001/14, and included most of the clade 2.3.4.4h isolates.

#### 3.2.5. PA Gene

**Figure 6:**
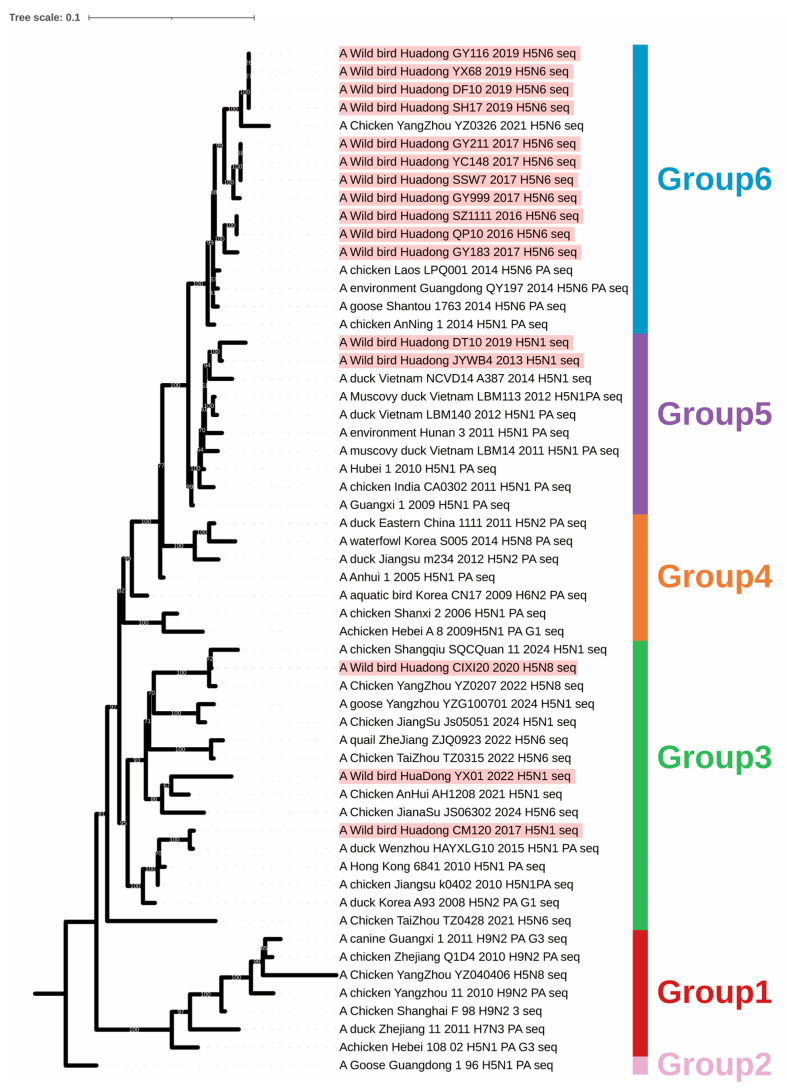
Phylogenetic tree based on the PA gene sequences of 16 H5 AIV isolates from wild birds in East China, 2013–2022. The isolates are highlighted with a pink background.

Phylogenetic analysis of the PA genes revealed six evolutionary clades (Figure 6). Group 1 was mainly derived from H9N2 subtype AIVs. The strains in Groups 2–6 all originated from A/goose/Guangdong/1/96. The PA genes of all H5N6 subtype isolates fell into Group 6. In Group 3, one PA gene originated from an H5N8 subtype AIV and two originated from H5N1 subtype AIVs; in Group 5, two PA genes originated from H5N1 subtype AIVs.

#### 3.2.6. NP Gene

**Figure 7:**
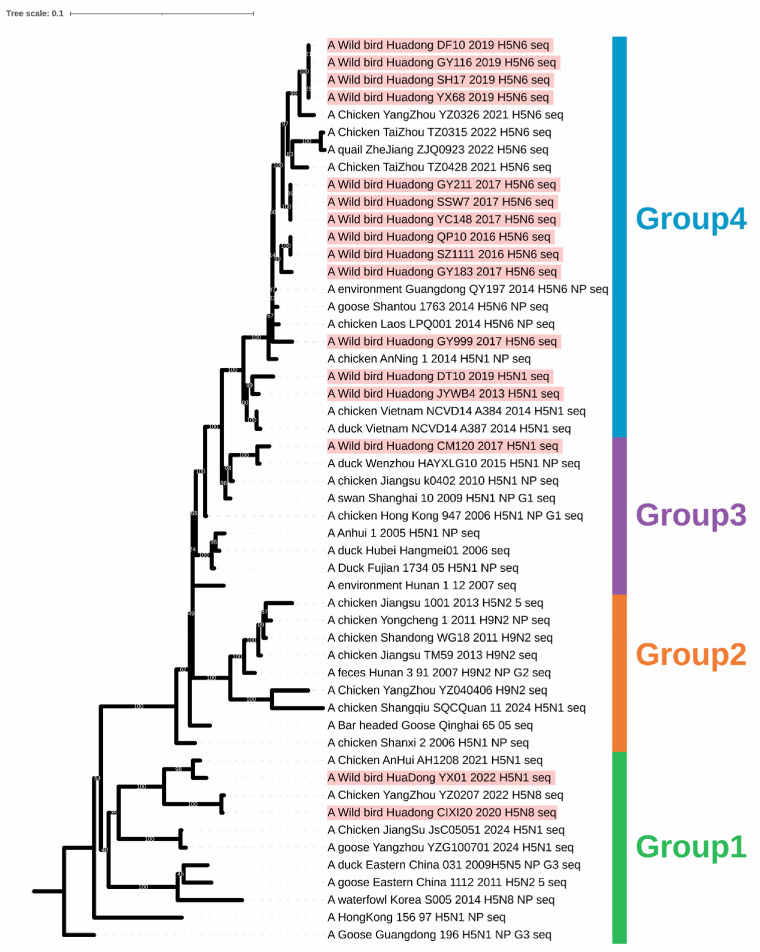
Phylogenetic tree based on the NP gene sequences of 16 H5 AIV isolates from wild birds in East China, 2013–2022. The isolates are highlighted with a pink background.

Phylogenetic analysis of the NP genes revealed four evolutionary clades (Figure 7). Group 1 originated from A/goose/Guangdong/1/96, and isolates belonging to clade 2.3.4.4b fell into this group. Group 4 was more closely related to clades 2.3.4.4d and 2.3.4.4h. These results indicated that the NP genes of the H5N6 subtype AIVs were predominantly clustered in Group 4.

#### 3.2.7. M Gene

**Figure 8:**
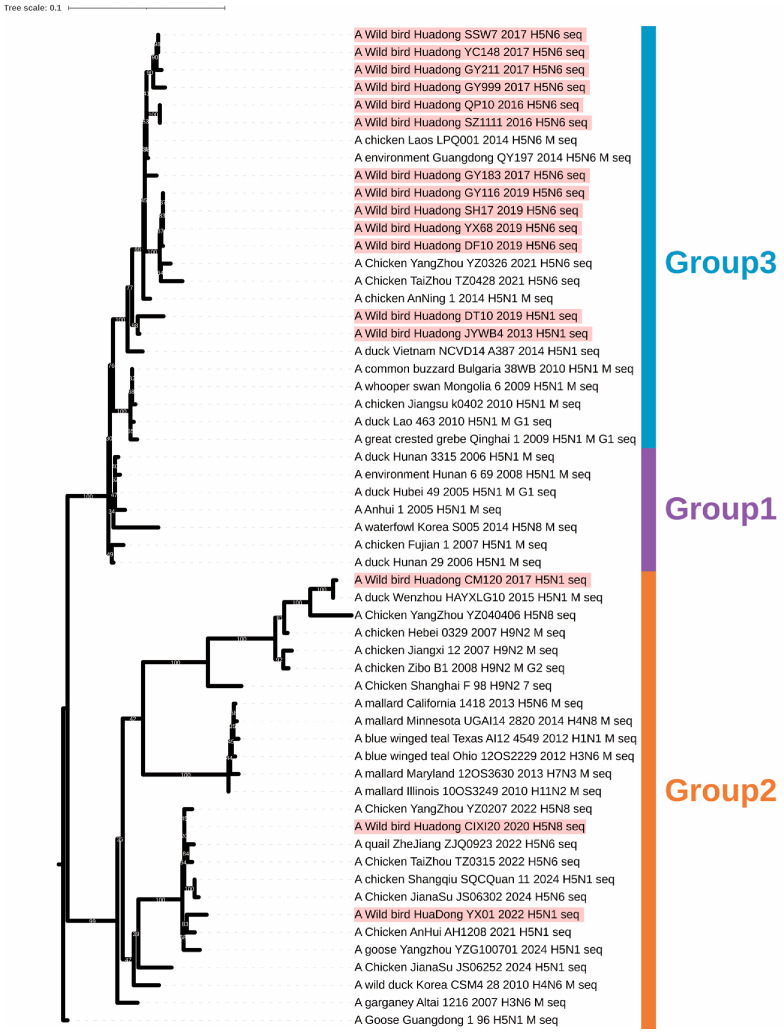
Phylogenetic tree based on the M gene sequences of 16 H5 AIV isolates from wild birds in East China, 2013–2022. The isolates are highlighted with a pink background.

Phylogenetic analysis of the M genes revealed two major clades (Figure 8): the Eurasian clade and the North American clade. All isolates in this study fell into the Eurasian clade, which was further subdivided into Groups 2 and 3. The M genes in Group 2 originated from H9N2 subtype AIVs prevalent in East China. The M genes of the vast majority of isolates fell into Group 3, and most of the isolates belonging to clades 2.3.4.4d and 2.3.4.4h were located in this group.

#### 3.2.8. NS Gene

**Figure 9:**
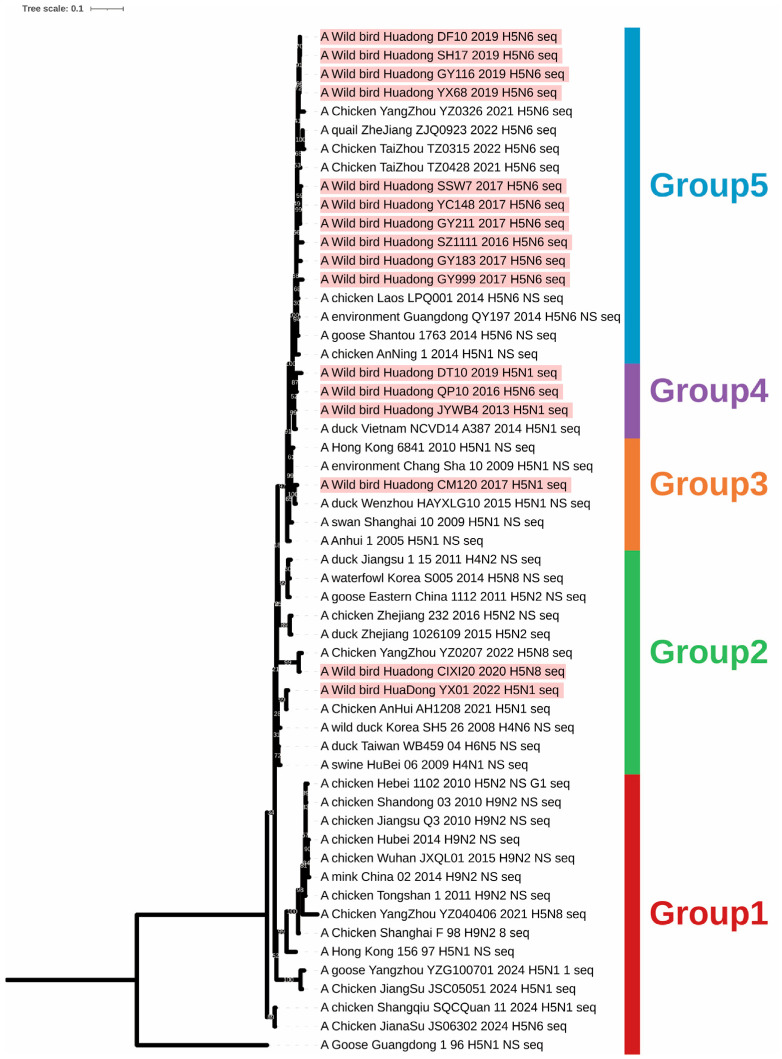
Phylogenetic tree based on the NS gene sequences of 16 H5 AIV isolates from wild birds in East China, 2013–2022. The isolates are highlighted with a pink background.

Phylogenetic analysis of the NS genes revealed several evolutionary clades (Figure 9). The clade 2.3.4.4b strains fell into Group 2, which originated from A/goose/Guangdong/1/96. Group 3 mainly originated from a clade 2.3.2.1e strain (A/duck/WenZhou/HAYXLG10/15) and included one H5N1 subtype AIV. Group 5 was closely related to a clade 2.3.4.4d strain (A/chicken/LPQ001/14), and most isolates belonging to clades 2.3.4.4d and 2.3.4.4h fell into this group.

### 3.3. Molecular Profiles

The HA cleavage site motifs of all 16 H5 subtype AIVs were either “PQRERRRKR↓GLF” or “PLRERRRKR↓GLF”, both of which contain multiple basic amino acids, indicating that all strains were highly pathogenic (Table 2). Analysis of the potential glycosylation sites of the HA genes of 16 H5 subtype isolates revealed that there were six conserved glycosylation sites at positions 27 (NSTE), 39 (NVTV), 180 (NNTN), 301 (NSSM), 498 (NGTY), and 557 (NGSL). Variations in glycosylation sites were also observed among different viral strains. A novel glycosylation site, NCSV, was identified at position 70 in Clade 2.3.4.4h isolates. Additional sites included NPTN or NPSN at position 100 and NHTS or NYTS at position 140 in some strains. In contrast, the glycosylation site NPTT at position 208 was absent in certain isolates (Table 3).

Analysis of potential glycosylation sites in the NA protein revealed two conserved sites at positions 146 (NGTV) and 235 (NGSC). The site at position 88 was absent only in the CM120 strain. Novel glycosylation sites emerged at positions 50, 58, 63, and 68 in the YX01 strain (Table 4). All H5N6 subtype avian influenza viruses (AIVs) possessed glycosylation sites at positions 51, 59, 134, 190, and 391, with some viruses acquiring an additional site, NPTT, at position 54. In clade 2.3.4.4h strains, the motif at position 51 changed from NETN to NDTS (Table 5). Glycosylation sites were consistently present at positions 54, 67, 84, and 144 on the NA protein of H5N8 subtype AIVs (Table 6).

In addition, multiple amino acid markers associated with pathogenicity, host adaptation, and antiviral resistance were identified in proteins including NA, PB2, PA, NP, M1, and NS1 (Table 7). The presence of these markers suggests that these wild bird-origin H5 subtype AIVs already possess a certain potential for mammalian adaptation, particularly the mutations in PB2 and NP, which warrant attention. Several pathogenicity-enhancing markers (e.g., PB2 K389R, NP Y52H, M1 N30D/T215A) indicate that these viruses have potential pathogenicity in mammals such as mice.

### 3.4. Gene Selection Pressure Analysis

Because the HA gene encodes the major surface antigen of influenza virus, directly mediates receptor binding, and plays a key role in immune escape, it is considered one of the gene segments under the strongest host immune selection pressure. Therefore, we performed selection pressure analysis on the HA gene sequences of 16 H5 subtype AIVs isolated from wild birds. The results showed that several sites, including positions 131, 136, 156, and 239, exhibited relatively high dN/dS values (Table 8). Notably, positions 131 and 156 showed relatively strong signals of positive selection in all three methods (MEME, FEL, and FUBAR). The positive selection sites detected in the vaccine strains were relatively limited, mainly sites 131, 136, and 205. In the reference strains, sites such as 10, 136, 156, 157, 171, 172, and 205 showed a certain degree of positive selection signals.

### 3.5. Antigenic Difference Analysis

**Figure 10:**
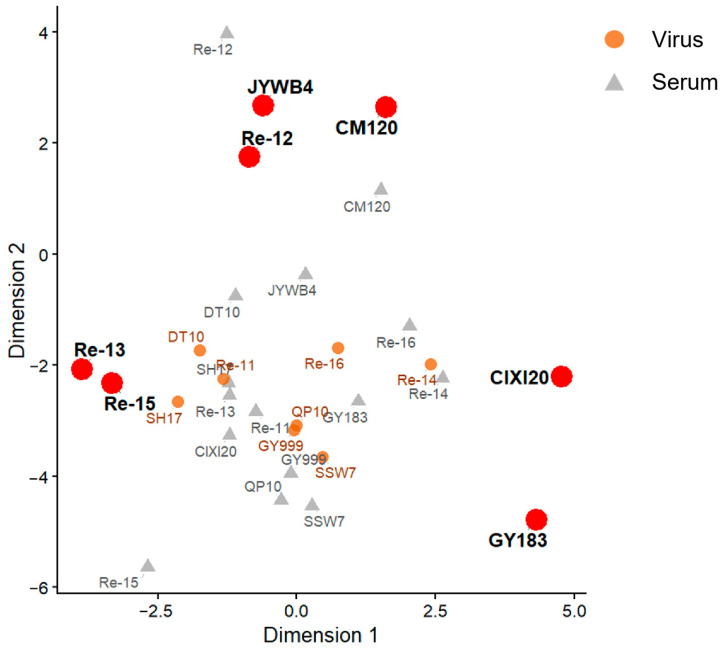
Antigenic cartography of H5 subtype AIVs. Orange: viral antigens; gray: sera; red-highlighted: strains most divergent from the main cluster.

Integrated analysis of the cross-hemagglutination inhibition (HI) assay (Supplementary Table S3) and antigenic cartography (Figure 10) revealed that the isolates and vaccine strains could be classified into multiple antigenic groups. Pairwise antigenic distances calculated from the antigenic map coordinates showed that the greatest distance was observed between GY183 and JYWB4 (8.94 antigenic units), followed by those between CIXI20 and Re-13 (8.65 antigenic units), GY183 and Re-13 (8.63 antigenic units), and GY183 and Re-12 (8.32 antigenic units) (Table 9). Accordingly, the strains exhibiting the most pronounced antigenic divergence were GY183 and CIXI20. Both viruses displayed large antigenic distances from the majority of the other strains, indicating substantial antigenic drift. The early epidemic strain GY183 was antigenically distant from the recent vaccine strains Re-12, Re-13, and Re-15 (ranging from 8.04 to 8.63 antigenic units), demonstrating a clear antigenic gap between the early isolate and the current vaccine strains. In contrast, DT10 and SH17 showed limited antigenic distances from their contemporaneous vaccine strain Re-11, suggesting that these isolates were antigenically well matched to the Chinese vaccine strains of the same period and that the vaccine strains could effectively cover the dominant circulating antigenic types at that time. However, CIXI20 was antigenically distant from its contemporaneous vaccine strain Re-12, Re-13 (6.87 and 8.65 antigenic units), indicating that antigenic mismatch may still exist between some circulating strains and the vaccines currently in use.

## 4. Discussion

Since the emergence of Gs/GD-lineage H5 viruses, H5 AIVs have diversified through clade turnover and frequent reassortment [22,23,24]. Rather than providing a comprehensive historical reconstruction, the present study offers a regional snapshot of H5 genetic and antigenic diversity in wild birds from East China between 2013 and 2022. The 16 isolates included H5N1, H5N6, and H5N8 viruses belonging to clades 2.3.2.1d, 2.3.2.1e, 2.3.4.4b, 2.3.4.4d, 2.3.4.4e, and 2.3.4.4h. The coexistence of these divergent HA clades, together with the heterogeneous phylogenetic origins of several internal gene segments, suggests that wild birds in this region may be exposed to multiple H5 lineages and reassortant gene pools. This is consistent with previous evidence that migratory wild birds can facilitate the movement and genetic mixing of avian influenza viruses across geographic regions [6,7,8]. These findings contribute to the understanding of H5 evolution in wild birds by showing that the East China segment of the East Asian–Australasian Flyway can serve as an important interface where genetically distinct H5 viruses are detected.

The detection of clade 2.3.4.4b viruses in 2020 and 2022 is noteworthy in the context of recent global H5N1/H5Nx evolution. Since 2020, clade 2.3.4.4b H5 viruses have become dominant in many regions and have been associated with extensive outbreaks in wild birds, poultry, and an increasing range of mammals [3,25,26]. The H5N8 isolate CIXI20/2020 and the H5N1 isolate YX01/2022 identified in this study are consistent with the broader replacement and geographic expansion of clade 2.3.4.4b viruses [25,26]. In contrast, the earlier H5N6 viruses detected in 2016–2019 belonged mainly to clades 2.3.4.4d, 2.3.4.4e, and 2.3.4.4h, reflecting the regional diversity of H5N6 viruses in East Asia before the widespread predominance of clade 2.3.4.4b [23,27] Therefore, our findings are broadly consistent with the global transition from multiple regionally circulating H5Nx lineages toward the increasing predominance of clade 2.3.4.4b.

All isolates contained multiple basic amino acids at the HA cleavage site, consistent with the molecular characteristics of highly pathogenic avian influenza viruses [22,28]. This finding indicates that highly pathogenic H5 viruses can be detected in wild birds in this region and may pose a risk to poultry populations if introduced into farms or live poultry markets. In addition to the cleavage motif, changes in HA and NA glycosylation patterns may influence receptor binding, antigenic exposure, and the balance between HA and NA functions [29,30,31]. The presence or loss of glycosylation sites near antigenic or receptor-binding regions, together with positively selected sites in HA, suggests that immune selection and host adaptation may contribute to the antigenic diversification of these viruses [5,32,33,34,35].

Several molecular markers associated with mammalian adaptation or increased pathogenicity were detected in PB2, PA, NP, M1, NA, and NS1 proteins. These markers have been reported to influence polymerase activity, host restriction, virulence in mammals, or host antiviral responses [12,13,14,15,16,17,18,19,20,21]. Their presence in wild-bird-origin H5 viruses deserves attention, especially in the context of the increasing global detection of clade 2.3.4.4b viruses in mammals [3,25].

Antigenic analysis is a critical approach for evaluating vaccine efficacy and the potential for viral immune escape, providing an antigenic basis for avian influenza vaccine strain selection, optimization of immunization programs, and epidemiological surveillance [36]. In this study, cross-hemagglutination inhibition (HI) assays revealed clear time-dependent antigenic drift among avian influenza isolates from different years: isolates from the same year exhibited limited antigenic distances, whereas those from different years showed pronounced antigenic divergence, a pattern driven primarily by high-frequency mutations in the HA gene and host immune selection [37,38]. The marked antigenic differences between early epidemic strains and recent vaccine strains reflect the continuous updating of vaccine strains in China. The majority of wild bird-derived isolates were antigenically well matched with the contemporary Chinese vaccine strains, indicating that these vaccines could effectively cover the dominant antigenic types circulating at the time. Notably, these vaccine strains not only conferred effective protection against viral circulation in domestic poultry in China but also displayed good antigenic matching with strains circulating in wild birds, underscoring a strong epidemiological linkage between wild bird and domestic poultry viruses [27,39]. Nevertheless, a small number of isolates still showed potential antigenic mismatch with the contemporary vaccine strains [26]. Therefore, continuous surveillance of avian influenza virus circulation in wild birds can provide early warning for domestic poultry, enabling timely detection of antigenic variants and guiding vaccine strain updates. This substantially improves early detection and helps reduce the risk of introduction and spread of the virus from wild birds to domestic poultry.

Several limitations should be considered when interpreting these findings. Although 27,000 samples were collected over a 10-year period, only 16 H5 viruses were isolated, and no isolates were obtained in several years. Therefore, the dataset is insufficient to estimate the true prevalence, annual continuity, or dominant evolutionary pathways of H5 AIVs in wild birds in East China. The antigenic analysis was based on HI assays and antigenic cartography, which provide valuable serological evidence but cannot fully predict vaccine protection in the field. Therefore, our findings should be interpreted as a surveillance-based snapshot of genetic and antigenic diversity rather than definitive evidence that wild birds maintain or drive H5 evolution in this region. Future studies should combine expanded longitudinal sampling, environmental and host-species metadata, poultry outbreak data, and in vivo vaccine-challenge experiments to clarify transmission pathways and vaccine relevance.

## 5. Conclusions

Our surveillance of wild birds in East China identified H5 subtype AIVs with genetic diversity, antigenic heterogeneity, and several molecular markers associated with mammalian adaptation. Although the number of isolates was limited, the detection of multiple subtypes and clades suggests that wild birds in this region may be involved in the regional movement and genetic diversification of H5 AIVs. These findings support the value of wild bird surveillance as a complementary component of avian influenza early warning and vaccine strain evaluation. However, this study has certain limitations. Although the surveillance spanned a decade, no viruses were isolated in some years, resulting in an uneven annual distribution that may affect the assessment of evolutionary continuity. Furthermore, the antigenic analysis was primarily based on in vitro HI assays and has not yet been validated by in vivo challenge experiments to assess immune protection. Future studies should integrate structural biology, serological surveillance, and cross-species transmission models to better understand the evolutionary dynamics and public health risks of H5 subtype AIVs.

## Figures and Tables

**Table 1:** Background of H5 AIV isolates in East China during 2013–2022.

Virus Isolate	Abbreviation	Sample Source	Subtype	Clade	GenBank Number (HA)
A/Wild bird/Huadong/JYWB4/2013	JYWB4	Wild bird	H5N1	2.3.2.1d	PZ417131
A/Wild bird/Huadong/QP10/2016	QP10	Wild bird	H5N6	2.3.4.4d	PZ417132
A/Wild bird/Huadong/SZ1111/2016	SZ1111	Wild bird	H5N6	2.3.4.4d	PZ417135
A/Wild bird/Huadong/CM120/2017	CM120	Wild bird	H5N1	2.3.2.1e	PZ417125
A/Wild bird/Huadong/GY183/2017	GY183	Wild bird	H5N6	2.3.4.4e	PZ417123
A/Wild bird/Huadong/GY211/2017	GY211	Wild bird	H5N6	2.3.4.4d	PZ417129
A/Wild bird/Huadong/GY999/2017	GY999	Wild bird	H5N6	2.3.4.4d	PZ417130
A/Wild bird/Huadong/SSW7/2017	SSW7	Wild bird	H5N6	2.3.4.4d	PZ417134
A/Wild bird/Huadong/YC148/2017	YC148	Wild bird	H5N6	2.3.4.4d	PZ417138
A/Wild bird/Huadong/DT10/2019	DT10	Wild bird	H5N1	2.3.2.1d	PZ417127
A/Wild bird/Huadong/DF10/2019	DF10	Wild bird	H5N6	2.3.4.4h	PZ417126
A/Wild bird/Huadong/GY116/2019	GY116	Wild bird	H5N6	2.3.4.4h	PZ417128
A/Wild bird/Huadong/SH17/2019	SH17	Wild bird	H5N6	2.3.4.4h	PZ417133
A/Wild bird/Huadong/YX68/2019	YX68	Wild bird	H5N6	2.3.4.4h	PZ417136
A/Wild bird/Huadong/CIXI20/2020	CIXI20	Wild bird	H5N8	2.3.4.4b	PZ417124
A/Wild bird/Huadong/YX01/2022	YX01	Wild bird	H5N1	2.3.4.4b	PZ417137

**Table 2:** Connecting-peptide at the cleavage site of HA.

Virus	Clade	Connecting-Peptide												
JYWB4/2013	2.3.2.1d	P	Q	R	E	R	R	R	K	R	↓	G	L	F
QP10/2016	2.3.4.4d	P	Q	R	E	R	R	R	K	R	↓	G	L	F
SZ1111/2016	2.3.4.4d	P	L	R	E	R	R	R	K	R	↓	G	L	F
CM120/2017	2.3.2.1e	P	Q	R	E	R	R	R	K	R	↓	G	L	F
GY183/2017	2.3.4.4e	P	L	R	E	R	R	R	K	R	↓	G	L	F
GY211/2017	2.3.4.4d	P	L	R	E	R	R	R	K	R	↓	G	L	F
GY999/2017	2.3.4.4d	P	L	R	E	R	R	R	K	R	↓	G	L	F
SSW7/2017	2.3.4.4d	P	L	R	E	R	R	R	K	R	↓	G	L	F
YC148/2017	2.3.4.4d	P	L	R	E	R	R	R	K	R	↓	G	L	F
DT10/2019	2.3.2.1d	P	Q	R	E	R	R	R	K	R	↓	G	L	F
DF10/2019	2.3.4.4h	P	L	R	E	R	R	R	K	R	↓	G	L	F
GY116/2019	2.3.4.4h	P	L	R	E	R	R	R	K	R	↓	G	L	F
SH17/2019	2.3.4.4h	P	L	R	E	R	R	R	K	R	↓	G	L	F
YX68/2019	2.3.4.4h	P	L	R	E	R	R	R	K	R	↓	G	L	F
CIXI20/2020	2.3.4.4b	P	L	R	E	K	R	R	K	R	↓	G	L	F
YX01/2022	2.3.4.4b	P	L	R	E	R	R	R	K	R	↓	G	L	F

**Table 3:** Predicted N-glycosylation sites in HA protein of H5 subtype viruses.

Virus	H5 Subtype									
	27	39	70	100	140	180	208	301	498	557
JYWB4/2013	NSTE	NVTV	-	-	-	NNTN	NPTT	NSSM	NGTY	NGSL
QP10/2016	NSTE	NVTV	-	-	-	NNTN	NPTT	NSSM	NGTY	NGSL
SZ1111/2016	NSTE	NVTV	-	-	-	NNTN	NPTT	NSSM	NGTY	NGSL
CM120/2017	NSTE	NVTV	-	-	-	NNTN	NPTT	NSSM	NGTY	NGSL
GY183/2017	NSTE	NVTV	-	-	-	NNTN	NPTT	NSSM	NGTY	NGSL
GY211/2017	NSTE	NVTV	-	-	NHTS	NNTN	NPTT	NSSM	NGTY	NGSL
GY999/2017	NSTE	NVTV	-	-	NHTS	NNTN	NPTT	NSSM	NGTY	NGSL
SSW7/2017	NSTE	NVTV	-	-	NHTS	NNTN	NPTT	NSSM	NGTY	NGSL
YC148/2017	NSTE	NVTV	-	-	NHTS	NNTN	NPTT	NSSM	NGTY	NGSL
DT10/2019	NSTE	NVTV	-	-	-	NNTN	NPTT	NSSM	NGTY	NGSL
DF10/2019	NSTE	NVTV	NCSV	-	NYTS	NNTN	NPTT	NSSM	NGTY	NGSL
GY116/2019	NSTE	NVTV	NCSV	-	NYTS	NNTN	NPTT	NSSM	NGTY	NGSL
SH17/2019	NSTE	NVTV	NCSV	-	NYTS	NNTN	NPTT	NSSM	NGTY	NGSL
YX68/2019	NSTE	NVTV	NCSV	-	NYTS	NNTN	NPTT	NSSM	NGTY	NGSL
CIXI20/2020	NSTE	NVTV	-	-	-	NNTN	NPTT	NSSM	NGTY	NGSL
YX01/2022	NSTE	NVTV	-	NPTN	-	NNTN	-	NSSM	NGTY	NGSL
Re-11	NSTE	NVTV	-	NPSN	NHTS	NNTN	NPTT	NSSM	NGTY	NGSL
Re-12	NSTE	NVTV	-	-	-	NNTN	NPTT	NSSM	NGTY	NGSL
Re-13	NSTE	NVTV	NCSV		NHTT	NNTN	NPTT	NSSM	NGTY	NGSL
Re-14	NSTE	NVTV	-	-	-	NNTN	NPTT	NSSM	NGTY	NGSL
Re-15	NSTE	NVTV	-	-	NHTS	NNTN	-	NSSM	NGTY	NGSL
Re-16	NSTE	NVTV	-	-	-	NNTN	NPTT	NSSM	NGTY	NGSL

**Table 4:** Predicted N-glycosylation sites in NA protein of H5N1 viruses.

Virus	H5N1 Subtype						
	50	58	63	68	88	146	235
JYWB4/2013	-	-	-	-	NSSL	NGTV	NGSC
CM120/2017	-	-	-	-	-	NGTV	NGSC
DT10/2019	-	-	-	-	NSSL	NGTV	NGSC
YX01/2022	NQSI	NNTW	NQTY	NISN	NSSL	NGTV	NGSC

**Table 5:** Predicted N-glycosylation sites in NA protein of H5N6 viruses.

Virus	H5N6 Subtype					
	51	54	59	134	190	391
QP10/2016	NETN	-	NITN	NGTI	NASA	NWSG
SZ1111/2016	NETN	-	NITN	NGTI	NASA	NWSG
GY183/2017	NETN	NPTT	NITN	NGTI	NASA	NWSG
GY211/2017	NETN	NPTT	NITN	NGTI	NASA	NWSG
GY999/2017	NETN	NPTT	NITN	NGTI	NASA	NWSG
SSW7/2017	NETN	NPTT	NITN	NGTI	NASA	NWSG
YC148/2017	NETN	NPTT	NITN	NGTI	NASA	NWSG
DF10/2019	NDTS	-	NITN	NGTI	NASA	NWSG
GY116/2019	NDTS	-	NITN	NGTI	NASA	NWSG
SH17/2019	NDTS	-	NITN	NGTI	NASA	NWSG
YX68/2019	NDTS	-	NITN	NGTI	NASA	NWSG

**Table 6:** Predicted N-glycosylation sites in NA protein of H5N8 viruses.

Virus	H5N8 Subtype			
	54	67	84	144
CIXI20/2020	NETV	NTSV	NNTE	NGTV

**Table 7:** Amino acid markers associated with specific phenotypic effects found in the genome of H5Nx viruses from wild birds.

Protein	Genetic Marker	Biological Functions
NA	deletion of 46–65 AA	Markers for enhanced pathogenicity and adaptation of the virus from wild birds to poultry [12]
PB2	L89V	Increased polymerase activity in mammalian cell lines and mice [13]
	G309D	Increased polymerase activity in mammalian cell lines and mice [13]
	K389R	Enhanced virulence of HPAIV H5 viruses in mice [14]
	R477G	Increased polymerase activity in mammalian cell lines and mice [13]
	M676T	Contributed to high replication and pathogenicity of influenza A virus in mammals [13]
PA	N409S	Increased polymerase activity in mammalian cell lines [15]
NP	Y52H	Confer resistance to inhibitors of BTN3A3 (butyrophilin subfamily 3 member A3) [16]
M1	N30D	Increased virulence in mice [17]
	T215A	
NS1	deletion of 80–84 AA	Decreased mammalian pathogenicity [18]
	W187R	Decreased mammalian pathogenicity [19]
	L103F	Increased virulence in mice [20]
	I106M	
	N205S	Decreased antiviral response in host [21]

**Table 8:** The selection profile of HA gene of H5 subtype viruses.

Virus	Site	MEME		FEL		FUBAR		SLAC	
		dN/dS	*p* Value	dN/dS	*p* Value	dN/dS	Posterior Probability	dN/dS	*p* Value
virus isolate	9	7.99	0.01	-	-	28.79	0.92	-	-
	131	6.30	0.02	5.88	0.02	126.35	0.98	-	-
	136	-	-	-	-	40.65	0.94	-	-
	156	3.27	0.09	3.27	0.07	133.32	0.98	-	-
	160	4.25	0.06	-	-	-	-	-	-
	167	4.00	0.06	-	-	-	-	-	-
	179	-	-	-	-	35.18	0.93	-	-
	239	3.82	0.07	-	-	43.93	0.94	-	-
	417	-	-	-	-	30.08	0.92	-	-
	420	4.82	0.04	-	-	-	-	-	-
vaccine strain	88	-	-	-	-	42.85	0.94	-	-
	131	7.97	0.008	8.02	0.005	962.55	0.99	3.62	0.04
	136	6.28	0.02	3.39	0.06	147.62	0.98	-	-
	149	3.56	0.08	-	-	-	-	-	-
	172	-	-	2.73	0.09	-	-	-	-
	204	3.87	0.07	-	-	-	-	-	-
	205	-	-	2.90	0.09	85.58	0.97	-	-
	342	10.02	0.003	-	-	-	-	-	-
reference strain	2	12.94	0.0007	-	-	-	-	-	-
	3	14.57	0.0003	-	-	-	-	-	-
	10	9.637	0.004	5.459	0.02	52.69	0.94	-	-
	14	7.75	0.009	-	-	-	-	-	-
	15	7.44	0.01	-	-	-	-	-	-
	52	11.96	0.001	3.03	0.08	-	-	-	-
	59	4.73	0.04	-	-	-	-	-	-
	82	4.53	0.05	-	-	-	-	-	-
	125	3.95	0.06	-	-	-	-	-	-
	136	6.70	0.02	-	-	42.47	0.92	5.48	0.09
	140	-	-	-	-	33.06	0.91	-	-
	154	3.94	0.07	-	-	-	-	-	-
	156	3.74	0.07	3.34	0.07	-	-	11.14	0.08
	157	5.32	0.03	5.26	0.02	34.19	0.91	3.00	0.09
	171	4.89	0.04	4.89	0.03	148.49	0.98	4.10	0.06
	172	6.72	0.02	6.74	0.01	119.43	0.97	5.00	0.02
	205	-	-	3.05	0.08	35.98	0.91	-	-
	210	-	-	2.82	0.09	-	-	-	-
	239	5.92	0.02	-	-	-	-	-	-
	273	3.42	0.09	3.42	0.06	-	-	-	-
	336	3.45	0.08	-	-	-	-	-	-
	348	3.34	0.09	-	-	-	-	-	-
	350	11.83	0.001	-	-	-	-	-	-
	416	3.43	0.09	-	-	-	-	-	-

**Table 9:** Antigenic distance between different strains (top 20).

Virus 1	Virus 2	Distance (Antigenic Units)
GY183	JYWB4	8.935648
CIXI20	Re-13	8.64905
GY183	Re-13	8.630393
GY183	Re-12	8.324965
CIXI20	Re-15	8.106754
GY183	Re-15	8.037562
GY183	CM120	7.911601
JYWB4	CIXI20	7.260305
CM120	Re-13	7.22475
CM120	Re-15	6.997874
SH17	CIXI20	6.935285
CIXI20	Re-12	6.868677
GY183	SH17	6.801959
GY183	DT10	6.794984
DT10	CIXI20	6.543541
SH17	CM120	6.499903
SSW7	JYWB4	6.421212
SSW7	CM120	6.406957
Re-13	Re-14	6.293727
GY183	Re-11	6.180426

## Data Availability

The original data presented in the study are openly available in GenBank under the accession numbers (Supplementary Table S2).

## Supplementary Materials

The following supporting information can be downloaded at https://www.mdpi.com/article/10.3390/ani16132109/s1. Table S1: Number of samples collected from each sampling location in East China, 2013–2022; Table S2: GenBank numbers of H5 AIVs isolates in East China during 2013–2022; Table S3: Cross HI titers(log2) of different viruses.

## Abbreviations

The following abbreviations are used in this manuscript:

AIV	Avian influenza virus
HI	Hemagglutination inhibition
HA	Hemagglutinin
HAU	Hemagglutination units
NA	Neuraminidase
SPF	Specific pathogen free
