# Supplementary material for: Marked Antigenic Divergence and Evolutionary Analysis of H5 AIVs from Wild Birds in East China, 2013–2022
Source: Animals (Basel). 2026 Jul 7;16(13):2109. doi: 10.3390/ani16132109 (PMC13359676; doi:10.3390/ani16132109)
Supplement: Supplementary file 1 [file animals-16-02109-s001.zip › Supplementary Table S1.pdf]

Supplementary Table S1. Number of samples collected from each sampling location in East China, 2013-2022.

**Sampling Location**	**Year**																	
	2013-2014		2014-2015		2015-2016		2016-2017		2017-2018		2018-2019		2019-2020		2020-2021		2021-2022	
	Fecal swabs	water samples	Fecal swabs	water samples	Fecal swabs	water samples	Fecal swabs	water samples	Fecal swabs	water samples	Fecal swabs	water samples	Fecal swabs	water samples	Fecal swabs	water samples	Fecal swabs	water samples
Suqian City, Jiangsu	130	10	100	10	450	20	300	20	470	20	465	10	420	30	475	20	230	10
Yancheng City, Jiangsu	130	10	150	10	640	20	630	20	750	20	670	10	540	30	550	20	500	10
Yangzhou City, Jiangsu	130	10	154	10	510	20	500	20	730	20	525	10	598	30	535	32	210	10
Wuxi City, Jiangsu	144	10	110	10	420	20	370	20	420	20	440	10	515	30	485	20	544	13
Suzhou City, Jiangsu	130	10	120	10	480	20	350	20	430	20	350	10	450	30	350	20	—	—
Qingpu District, Shanghai	100	10	100	10	450	20	200	20	370	20	500	10	450	20	450	20	310	10
Chongming District, Shanghai	100	10	100	10	200	20	350	20	450	20	350	10	365	30	480	20	300	10
Ningbo City, Zhejiang	100	10	100	10	250	20	200	20	300	20	370	10	520	30	500	30	230	10
**Total samples**	1044		1014		3560		3060		4080		3750		4088		4007		2397	
