# Supplementary material for: Marked Antigenic Divergence and Evolutionary Analysis of H5 AIVs from Wild Birds in East China, 2013–2022
Source: Animals (Basel). 2026 Jul 7;16(13):2109. doi: 10.3390/ani16132109 (PMC13359676; doi:10.3390/ani16132109)
Supplement: Supplementary file 1 [file animals-16-02109-s001.zip › Supplementary Table S2.pdf]

Supplementary Table S2. GenBank numbers of H5 AIVs isolates in East China during 2013-2022.

Virus isolate	GenBank number							
	PB2	PB1	PA	HA	NP	NA	M	NS
A/Wild bird/Huadong/JYWB4/2013	PZ418684	PZ418700	PZ418718	PZ417131	PZ418739	PZ417633	PZ419518	PZ419541
A/Wild bird/Huadong/QP10/2016	PZ418685	PZ418701	PZ418719	PZ417132	PZ418740	PZ417634	PZ419519	PZ419542
A/Wild bird/Huadong/SZ1111/2016	PZ418688	PZ418704	PZ418722	PZ417135	PZ418743	PZ417637	PZ419522	PZ419545
A/Wild bird/Huadong/CM120/2017	PZ418678	PZ418694	PZ418712	PZ417125	PZ418733	PZ417627	PZ419512	PZ419535
A/Wild bird/Huadong/GY183/2017	PZ418676	PZ418692	PZ418710	PZ417123	PZ418731	PZ417625	PZ419510	PZ419533
A/Wild bird/Huadong/GY211/2017	PZ418682	PZ418698	PZ418716	PZ417129	PZ418737	PZ417631	PZ419516	PZ419539
A/Wild bird/Huadong/GY999/2017	PZ418683	PZ418699	PZ418717	PZ417130	PZ418738	PZ417632	PZ419517	PZ419540
A/Wild bird/Huadong/SSW7/2017	PZ418687	PZ418703	PZ418721	PZ417134	PZ418742	PZ417636	PZ419521	PZ419544
A/Wild bird/Huadong/YC148/2017	PZ418691	PZ418707	PZ418725	PZ417138	PZ418746	PZ417640	PZ419525	PZ419548
A/Wild bird/Huadong/DT10/2019	PZ418680	PZ418696	PZ418714	PZ417127	PZ418735	PZ417629	PZ419514	PZ419537
A/Wild bird/Huadong/DF10/2019	PZ418679	PZ418695	PZ418713	PZ417126	PZ418734	PZ417628	PZ419513	PZ419536
A/Wild bird/Huadong/GY116/2019	PZ418681	PZ418697	PZ418715	PZ417128	PZ418736	PZ417630	PZ419515	PZ419538
A/Wild bird/Huadong/SH17/2019	PZ418686	PZ418702	PZ418720	PZ417133	PZ418741	PZ417635	PZ419520	PZ419543
A/Wild bird/Huadong/YX68/2019	PZ418689	PZ418705	PZ418723	PZ417136	PZ418744	PZ417638	PZ419523	PZ419546
A/Wild bird/Huadong/CIXI20/2020	PZ418677	PZ418693	PZ418711	PZ417124	PZ418732	PZ417626	PZ419511	PZ419534
A/Wild bird/Huadong/YX01/2022	PZ418690	PZ418706	PZ418724	PZ417137	PZ418745	PZ417639	PZ419524	PZ419547
