# Supplementary material for: Marked Antigenic Divergence and Evolutionary Analysis of H5 AIVs from Wild Birds in East China, 2013–2022
Source: Animals (Basel). 2026 Jul 7;16(13):2109. doi: 10.3390/ani16132109 (PMC13359676; doi:10.3390/ani16132109)
Supplement: Supplementary file 1 [file animals-16-02109-s001.zip › Supplementary Table S3.pdf]

Supplementary Table S3. Cross HI titers(log2) of different viruses.

Virus	Serum														
	JYWB4	QP10	GY183	GY999	SSW7	CM120	SH17	DT10	CIXI20	Re-11	Re-12	Re-13	Re-14	Re-15	Re-16
JYWB4	8.33 ± 0.58	6 ± 0	2.33 ± 0.58	6.33 ± 0.58	5 ± 1	6.33 ± 0.58	4.67 ± 0.58	6.67 ± 0.58	4 ± 1	6.67 ± 0.58	8 ± 0	2.33 ± 0.58	6 ± 1	5 ± 0	7.33 ± 0.58
QP10	5 ± 0	11.33 ± 0.58	5.67 ± 0.58	11.33 ± 0.58	11.33 ± 0.58	4.67 ± 0.58	9 ± 0	9.33 ± 0.58	5 ± 0	10.33 ± 0.58	6 ± 0	7 ± 0	8 ± 0	6 ± 0	8.67 ± 0.58
GY183	3.33 ± 0.58	8 ± 1	6 ± 0	7 ± 0	7 ± 0	3 ± 1	5.33 ± 0.58	5 ± 0	7.33 ± 0.58	7.33 ± 0.58	2 ± 1	4.33 ± 0.58	6.67 ± 0.58	4 ± 1	8 ± 0
GY999	5 ± 0	10.67 ± 0.58	6.33 ± 0.58	10.33 ± 0.58	10.33 ± 0.58	2.67 ± 0.58	8.33 ± 0.58	8.33 ± 0.58	5 ± 0	9 ± 0	7.33 ± 0.58	6 ± 0	7 ± 0	6 ± 0	9.33 ± 0.58
SSW7	2.67 ± 0.58	9 ± 0	5 ± 0	7.67 ± 0.58	9.67 ± 0.58	1.67 ± 0.58	6 ± 1	5 ± 1	5.33 ± 0.58	7 ± 0	2 ± 0	7.33 ± 0.58	7.67 ± 0.58	6 ± 1	9 ± 0
CM120	7.33 ± 0.58	7 ± 1	3 ± 1	7.33 ± 0.58	5.33 ± 0.58	10.67 ± 0.58	6 ± 0	7 ± 0	6 ± 0	7 ± 1	9 ± 0	4 ± 0	8.33 ± 0.58	5 ± 0	9 ± 1
SH17	4 ± 0	8 ± 0	3 ± 1	7 ± 0	6 ± 0	4 ± 0	9 ± 0	9 ± 0	4 ± 0	9 ± 0	4.33 ± 0.58	7.33 ± 0.58	5 ± 0	5.33 ± 0.58	6.33 ± 0.58
DT10	7 ± 0	6.67 ± 0.58	3.67 ± 0.58	7 ± 0	7.33 ± 0.58	5 ± 0	9.33 ± 0.58	10.33 ± 0.58	4.67 ± 0.58	7.67 ± 0.58	9.33 ± 0.58	8 ± 1	7 ± 0	6.33 ± 0.58	8.33 ± 0.58
CIXI20	2.33 ± 0.58	8.67 ± 0.58	4.33 ± 0.58	8.33 ± 0.58	8 ± 0	2.67 ± 0.58	9 ± 0	8 ± 0	3 ± 0	8.33 ± 1.15	3.33 ± 0.58	6 ± 0	3.67 ± 0.58	7 ± 0	5.67 ± 0.58
Re-11	3.33 ± 0.58	9 ± 0	4 ± 0	8 ± 0	8.33 ± 0.58	2.67 ± 0.58	8.67 ± 0.58	7.67 ± 0.58	2.67 ± 0.58	7.33 ± 0.58	5 ± 1	6.33 ± 0.58	5.67 ± 0.58	6.67 ± 0.58	8 ± 0
Re-12	8 ± 0	2 ± 0	0 ± 0	1.33 ± 0.58	1.67 ± 1.15	4 ± 0	0 ± 0	2.67 ± 0.58	0 ± 0	3 ± 1	9 ± 0	0 ± 0	0 ± 0	3.33 ± 0.58	5.33 ± 0.58
Re-13	5 ± 0	10.33 ± 0.58	6.33 ± 0.58	9.67 ± 0.58	10 ± 0	7.33 ± 0.58	11.33 ± 0.58	11 ± 0	4 ± 0	10 ± 0	6.67 ± 0.58	10.33 ± 0.58	8.33 ± 0.58	8 ± 0	8 ± 0
Re-14	5.33 ± 0.58	10 ± 0	6.33 ± 0.58	9 ± 0	10.33 ± 0.58	5.67 ± 0.58	6 ± 1	7.33 ± 0.58	10.33 ± 0.58	8 ± 0	6.33 ± 0.58	4.33 ± 0.58	11.67 ± 0.58	7.33 ± 0.58	10.67 ± 0.58
Re-15	2 ± 1	8.33 ± 0.58	3.33 ± 0.58	7.67 ± 0.58	6.67 ± 0.58	2 ± 0	8 ± 0	8 ± 0	3.67 ± 0.58	9.33 ± 0.58	5 ± 0	5 ± 0	6 ± 0	11.67 ± 0.58	10.33 ± 0.58
Re-16	7 ± 0	9 ± 0	5 ± 0	6.67 ± 0.58	8 ± 0	3.67 ± 0.58	4 ± 0	5.67 ± 0.58	7.33 ± 0.58	5.67 ± 0.58	7.33 ± 0.58	5.33 ± 0.58	10.67 ± 0.58	8 ± 0	10.67 ± 0.58

Values are presented as mean ± SD from three independent replicates (n=3).
